# Response Letter: Interpreting Echocardiographic Right Ventricular–Pulmonary Arterial Coupling Indices After Transcatheter Tricuspid Intervention

**DOI:** 10.1016/j.shj.2026.100845

**Published:** 2026-05-08

**Authors:** Jennifer von Stein, Christos Iliadis

**Affiliations:** Department III of Internal Medicine, Faculty of Medicine and University Hospital Cologne, University of Cologne, Cologne, Germany; Clinical Trials Center, Cardiovascular Research Foundation, New York, New York, USA; Center for Cardiovascular Medicine ABCD, Aachen – Bonn – Cologne – Düsseldorf, Germany; Department III of Internal Medicine, Faculty of Medicine and University Hospital Cologne, University of Cologne, Cologne, Germany; Center for Cardiovascular Medicine ABCD, Aachen – Bonn – Cologne – Düsseldorf, Germany

We would like to thank Pagnoni et al. for their thoughtful comments and for highlighting important aspects regarding the interpretation of echocardiographic right ventricular–pulmonary arterial coupling (RVPAC) indices following transcatheter tricuspid valve repair (TTVr). RVPAC represents a fundamental physiological concept describing the interaction between right ventricular contractility and pulmonary arterial afterload, traditionally derived from pressure–volume loop analysis as the ratio of end-systolic elastance to arterial elastance.

In the presence of significant tricuspid regurgitation (TR), RVPAC indices may be influenced by the redistribution of right ventricular stroke volume between the pulmonary circulation and the right atrium, affecting the estimation of pulmonary artery systolic pressure (PASP) and thereby altering coupling metrics.^1^ Following TTVr, restoration of forward flow may lead to apparent changes in RVPAC that reflect altered loading conditions rather than intrinsic deterioration of right ventricular function.

Consistently, in patients undergoing TTVr, a decline in tricuspid annular plane systolic excursion (TAPSE)/PASP following intervention has been associated with improved clinical outcomes, supporting the concept that changes in coupling after valve repair should be interpreted in light of the underlying hemodynamic shifts rather than as a marker of worsening right ventricular performance.^2^

Although echocardiographic indices approximating RVPAC, such as TAPSE/PASP, remain load-dependent, their integration has consistently demonstrated prognostic relevance across a broad range of cardiovascular conditions, predominantly based on resting assessment.^3^ However, conventional echocardiographic parameters and integrated measures such as the myocardial performance index and contractility index (dp/dt), are generally more load-dependent and less practical for clinical use. Thus, TAPSE/PASP offers a favorable balance between relative load-independence and feasibility in clinical practice.

In parallel, efforts have been made to develop alternative, more physiologically grounded surrogates of RVPAC. A volumetric-based index, defined as the ratio of forward stroke volume to right ventricular end-systolic volume, has been invasively validated in patients with pulmonary hypertension and shown to closely reflect RVPAC.^4^ In patients with TR, this volumetric approach may be particularly relevant, as it inherently accounts for regurgitant volume and altered loading conditions.

In this context, we have recently investigated volumetric RVPAC in patients undergoing TTVr, including its temporal changes following intervention, and observed that it provides improved prognostic discrimination compared with conventional coupling metrics (*JACC: Cardiovascular Interventions*. In press). Forward stroke volume increased in patients with effective TR reduction, while right ventricular end-systolic volume remained unchanged, resulting in an increase in this ratio. However, these early changes were not independently associated with clinical outcomes, indicating that short-term alterations in RVPAC primarily reflect changes in loading conditions rather than intrinsic right ventricular function.

Taken together, we agree that a more comprehensive assessment of RVPAC, including longitudinal evaluation, may further refine its interpretation. In particular, the long-term evolution of RVPAC following TTVr remains insufficiently characterized. Although exercise-based assessment may provide additional physiological insights^5^ and potentially unmasks impaired RV–PA coupling, its clinical applicability and interpretation remain complex and not yet well established. In particular, stress-based assessment is subject to methodological variability and limited standardization, and its interpretation may be further complicated in the presence of significant TR. We further agree that functional capacity and exercise tolerance are important outcomes that may complement echocardiographic coupling indices and refine assessment of clinical response after TTVr, with quality of life representing a particularly relevant outcome in older and comorbid populations.

## Funding

The authors have no funding to report.

## Disclosure Statement

Jennifer von Stein has received lecture fees from Edwards Lifesciences. Christos Iliadis has received travel support and consultant honoraria by Abbott and Edwards Lifesciences.
